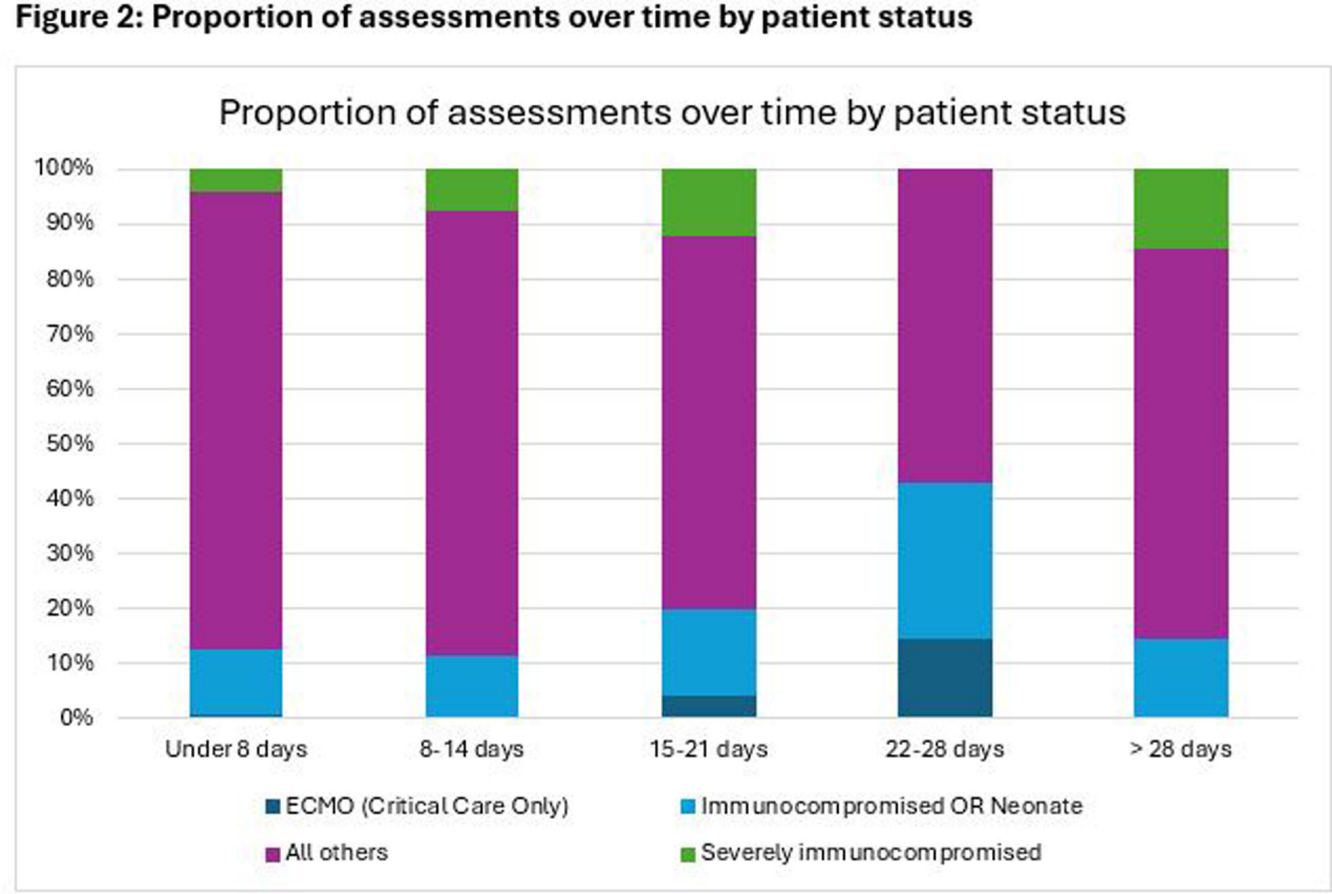# 365 Beyond the Patient: The Hidden Epidemiological Footprint of Recombinant Gene Therapy

**DOI:** 10.1017/ash.2026.10701

**Published:** 2026-06-23

**Authors:** Christelle Ilboudo, Kathlyn Baharaeen, Jennifer Heltzer, Elizabeth Monsees

**Affiliations:** 1 Children’s Mercy Kansas City; 2 Children’s Mercy Hospital

## Abstract

**Background:** Seasonal viral respiratory pathogens increase isolation needs in pediatric care, creating workflow challenges. A survey found variation in isolation guidelines, particularly regarding duration. In Phase 1, we focused on aligning isolation orders, signage, and infection prevention auditing time. In Phase 2, we sought to implement a nurse-led de-isolation process, empowering nurses to evaluate symptoms and determine readiness to discontinue isolation precautions. **Methods:** An interdisciplinary quality improvement team mapped isolation work processes, identifying 7 decision points for initiating or discontinuing isolation. Using a prioritization matrix, we developed an EMR-integrated, nurse-driven de-isolation tool (figure 1) that generated a nursing task every 7-28 days based on patient status due to protracted illness (e.g., immunocompromised, severely immunocompromised, or neonates). Predefined clinical criteria guided assessments, triggering discontinuation when met, and eliminating clinician intervention. Patients on Extracorporeal Membrane Oxygenation (ECMO) were excluded. We used iterative cycles of testing and refinement to continuously improve the automation process and clarify response options. Outcome measures included successful discontinuation of isolation precautions; process measures surveyed for nursing satisfaction and assessed appropriateness of decisions based on IPC audits; and balancing measures monitored hospital-acquired viral respiratory infections. Data from a 6-month period were analyzed using descriptive statistics. **Results:** Before implementation, 39 nurses tested the tool. The majority reported satisfaction with its ease of use, clarity of clinical definitions, and appropriateness of assessment outcomes. Completion time ranged from 20 seconds to 5 minutes. 310 nursing assessments were analyzed; 34% discontinued isolation precautions, 50% maintained precautions with discussion during rounds, and the remainder involved patients automatically retained in isolation due to their special status (neonates, immunocompromised) (Table 1). We identified that 72 assessments during the refinement process misfired without an active isolation order. Then, among the 238 assessments with order and assessment dates, the median time between order placement and nursing assessment was 7 days, with an average of 10 days. Most assessments occurred within 14 days for patients without protracted illness status. Over time, the proportion of patients with protracted illness status increased (Figure 2). No clusters of hospital-acquired viral infections were observed, and IP audits confirmed agreement with the nurses’ assessment in most cases. **Conclusion:** In this phase, we revealed that a nurse-driven, patient-tailored de-isolation process created a systematic approach to remove isolation without increasing exposure risk. Future opportunities entail strengthening congruency between IP and nursing assessment and de-isolation decisions for patients with underlying clinical conditions who may have a more protracted illness.

**Figure:**
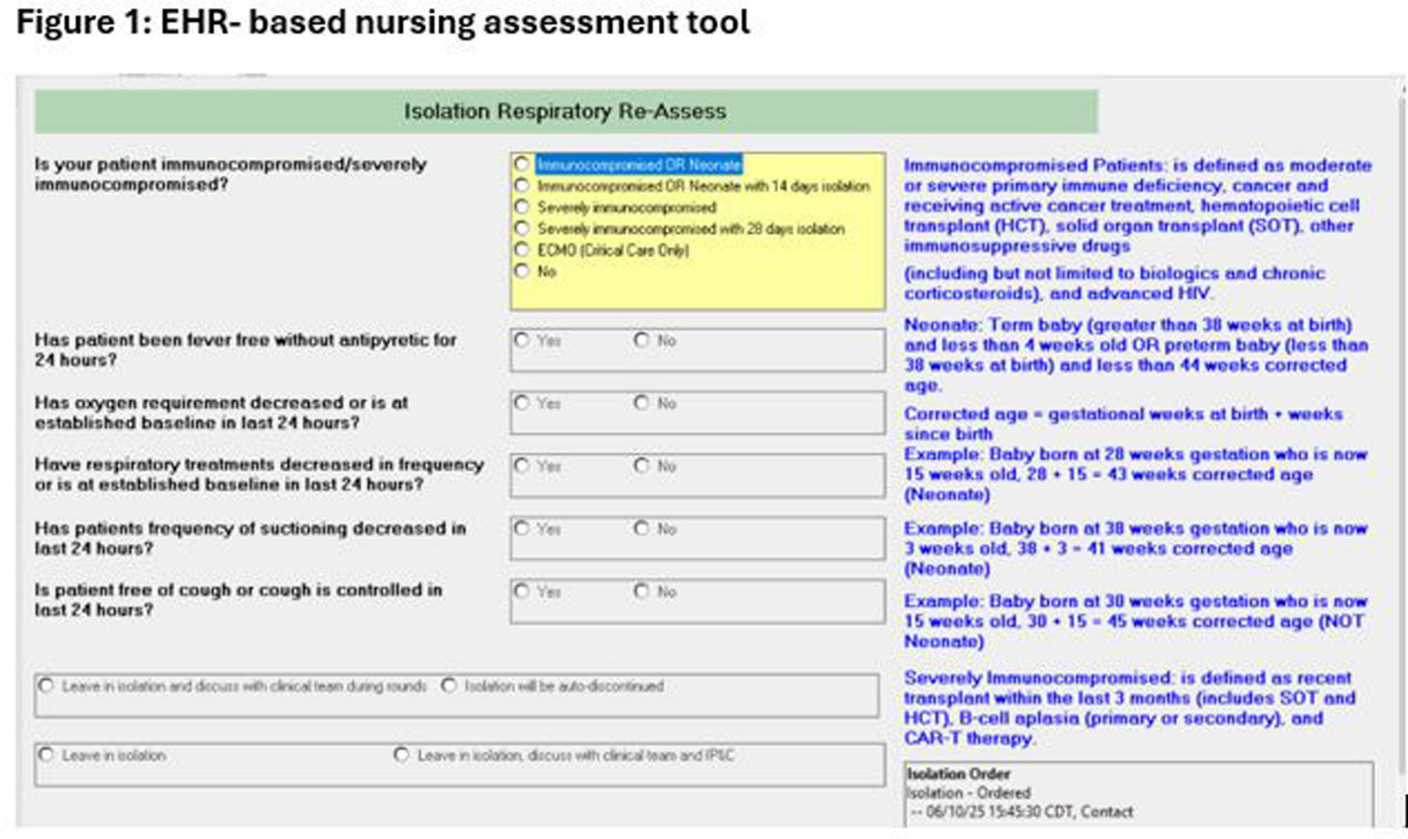

**Figure:**
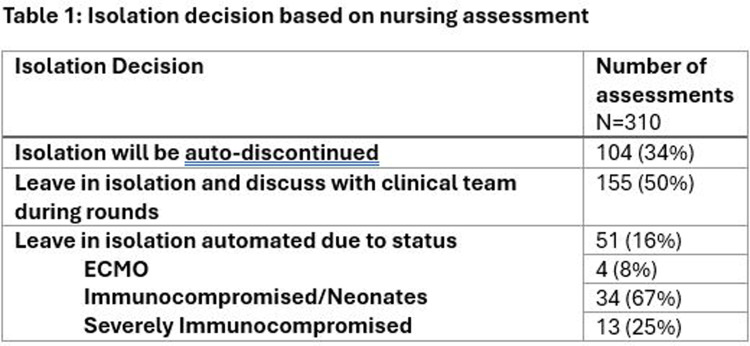

**Figure:**